# Consumers’ Preference for the Consumption of the Fresh Black Slavonian Pig’s Meat

**DOI:** 10.3390/foods12061255

**Published:** 2023-03-15

**Authors:** Sanja Jelić Milković, Ružica Lončarić, Igor Kralik, Jelena Kristić, Ana Crnčan, Ivona Djurkin Kušec, Maurizio Canavari

**Affiliations:** 1 Faculty of Agrobiotechnical Sciences Osijek, Josip Juraj Strossmayer University of Osijek, Vladimira Preloga 1, 31000 Osijek, Croatia; 2 Department of Agricultural and Food Sciences, Alma Mater Studiorum-University of Bologna, Viale Giuseppe Fanin 50, 40126 Bologna, Italy

**Keywords:** consumer preferences, fresh meat, Black Slavonian pig, information treatment, choice experiment, willingness to pay

## Abstract

There are limited data on Croatian consumers’ preferences and willingness to pay for fresh meat from the Black Slavonian pig. The survey was conducted on a sample of *n* = 410 Croatian consumers, using a hypothetical choice experiment to examine how food attributes and information treatment influence consumers’ decisions regarding fresh ham meat of the Black Slavonian pig. The hypothetical choice experiment was conducted using fresh boneless pork ham with three attributes (price, colour and geographical information) as the focuses of the product. Croatian consumers were randomly assigned to one of two treatment options (control or information) in an online survey to investigate the role of information. Our results indicate that Croatian consumers have a clear preference for fresh boneless ham from the Black Slavonian pig produced in both production systems and labelled as reared in continental Croatia and with a protected designation of origin (PDO) label, compared with fresh boneless ham produced from conventionally reared pigs (intensive rearing—indoor rearing) without the label. The results show that the information given to consumers about the production system, meat colour and geographical information positively influenced consumer choice. Appropriate labelling and information about the product can positively influence consumers’ preferences, which indicates the importance of highlighting the traditional characteristics (production system, darker colour of the meat and production area) of fresh meat from Black Slavonian pig on the label in promotional activities.

## 1. Introduction

The share of animal production in the total value of agricultural production in the Republic of Croatia in 2020 is 38.5%, while the share of crop production in total agricultural production is 61.5% [1]. Pig production in the Republic of Croatia is a significant branch of livestock production for supplying the market with high-quality meat [2]. Croatia has a long tradition of pork production organised in family farms and large pig farms [3,4], mainly in intensive production systems. However, intensive pig rearing systems are becoming increasingly unacceptable due to general environmental issues, animal welfare, farm-animal suffering and stress, and pain management [5]. Meat production and consumption are associated with significant environmental impacts and pollution, as well as resource inefficiencies [6]. According to Djekic and Tomasevic [7], meat production and consumption impact three pillars of sustainability (economic, social and environmental), and meat is considered the food with the greatest impact on the entire food supply chain, with the greatest impact in the livestock sector, which has the highest environmental impact. The pig sector is the largest contributor to global meat production at over 37%, with global meat production expected to increase by over 37% and global demand for pork by over 35% by 2030 [7]. All environmental impacts in the meat supply chain originate on the farm, while only 12% are related to post-farm activities, such as processing, transport and retail [8]. The FAO has recently introduced a new term, “sustainable diets”, that focuses on diets with optimal health and low environmental impact, linking environmental impact to production and consumption. Based on a conducted Monte Carlo simulation, Djekic et al. [8] conclude that the average Serbian pork consumer is responsible for emitting about 4 kg CO_2_-eq per week. Daily meat consumption is responsible for emissions of 2.80 kg CO_2_-eq per capita, with beef contributing to 65% of emissions [6]. Intensive pork production emits 668 million tonnes of CO_2_-eq of greenhouse gases every year and is considered one of the largest polluters [9]. Therefore, it is now essential in livestock production to develop alternative systems that balance sustainability and food security [6]. Sustainability, environmental, economic and social concerns play an important role in shaping the EU meat market [10]. In addition, consumers’ concerns about the environment and climate change are leading them to pay more attention to production processes (local markets, organic and other quality systems, animal welfare and environmental footprint) [10]. All these changes in the market, consumer behaviour and the European Commission’s forecasts are in line with the initiatives to promote local pig production.

The Black Slavonian pig is an indigenous Croatian pig breed. Indigenous and protected pig breeds represent the genetic heritage of a country, and the breeding of local indigenous breeds aims to preserve biodiversity, which is important from a historical, economic and cultural perspective [11]. Rearing of this breed is more economical and profitable compared with cosmopolitan breeds due to their longevity, resilience and adaptability to extensive rearing conditions [12]. Additionally, rearing pigs in traditional free-range systems is also cost and labour-efficient as well as environmentally and animal friendly, resulting in better meat quality traits and nutritional characteristics [13,14,15,16,17]. Due to these advantages over modern pig breeds, the meat of this breed has recently been protected at the national level with the protected designation of origin (PDO) label (EU level pending) [18]. Products from indigenous breeds have high gastronomic and economic characteristics, such as unique quality, environmental benefits, and cultural and ethnic merits, which consumers appreciate and value [19,20]. Consumers are the last step in the production chain, so it is important to understand the factors that influence consumer behaviour [21]. Consumer preferences, behaviour and perceptions of meat and meat products are heterogeneous. They depend not only on the appearance and sensory characteristics of the product, but also on psychological (beliefs, attitudes, expectations, lifestyle and values), marketing (price, label, availability) and environmental aspects [21,22]. Understanding the factors that influence consumer perceptions of a product’s value or cost is critical to product innovation, marketing strategy choice and maintaining a competitive advantage [23]. It is difficult for consumers to form quality expectations, especially for fresh meat about which little information is available. According to research, expected quality appears to be one of the most important factors in consumers’ food purchase intention [21]. Quality cues can be intrinsic (colour, fat content, marbling) and extrinsic (price, origin, quality label). Their role in developing expectations depends on the type of meat or meat products and the circumstances in which the product is used or consumed. In addition, quality attributes such as traditional food, health, naturalness, sustainability or environmental benefits cannot be directly assessed but are nonetheless part of the benefits expected by consumers [21,24,25].

Previous research on pork products in the Republic of Croatia has been mainly concerned with consumer preferences regarding the purchase of organic or conventional pork products, frequency of purchase, place of purchase and satisfaction with the offer of such products. Few studies have examined Croatian consumers’ preferences for social and altruistic attributes of pork products, animal welfare and sustainability [26,27,28,29]. However, with better insight into consumer preferences for production attributes, it is possible to develop new markets between traditional and organic production, focusing on product attributes related to animal welfare, traceability and health products [30]. Croatian consumers are not yet sufficiently familiar with the impact of animal welfare on meat quality, but their awareness is growing, and they are increasingly demanding transparency in food production [31]. It is important to provide consumers with information to improve their knowledge of how animal welfare is ensured on farms and in slaughterhouses, to make them aware of the importance of humane treatment of animals and to show the impact of animal treatment on meat quality. Information about consumers’ wants and needs should be important for producers so that they can provide products for which consumers are willing to pay. Their goal should therefore be to have the right products for the right customers [30,32].

Our study firstly contributes to the literature as the methodology presented can be applied to other food products to study consumer heterogeneity and predict consumer behaviour, which is one of the biggest challenges in marketing. Secondly, to our knowledge, this is the only study that, by including the effect of information in the choice experiment, evaluates how information about the production system, indirectly related to animal welfare, environmental protection and biodiversity conservation, positively affects consumer preference and willingness to pay a premium price for fresh meat from the Black Slavonian pig.

## 2. Materials and Methods

This section introduces the chosen product attributes and levels, explains the used treatments and describes the choice experiment design, survey and estimation procedure.

### 2.1. Explanation of Treatments and Selection of Choice Attributes and Levels

A hypothetical choice experiment was used to investigate participants’ preferences and willingness to pay (WTP) for the fresh meat of Black Slavonian pigs reared outdoors and semi-indoors. By leveraging the lack of information and knowledge on the fresh meat of Black Slavonian pigs, this research focused on the effect of information on consumer preferences. Using treatments, we evaluate whether information about the product could positively affect consumer opinion about the product and WTP. Choice experiments simulate a real-life retail environment where consumers choose between products with similar attributes and allow researchers to estimate trade-offs among alternatives [33,34]. In this research, the different fresh meat products differed in terms of production system, colour, geographical information and price. These attributes were selected based on the specifications for obtaining a PDO mark [35] and the literature review [19,36,37,38,39,40]. Table 1 shows the attribute and attribute levels evaluated in the choice experiment.

The choice experiment was conducted using prices expressed in Croatian kuna (HRK), and the price attribute included three levels: HRK 70.00 (€9.24), HRK 120.00 (€15.85) and HRK 170.00 (€22.45) per kilogram, which were converted into euros for the purposes of this article. Prices chosen for the choice experiment reflect market prices for one kilogram of fresh pork ham without bones (*Gluteus Medius*) originating from Black Slavonian pigs at the time of the study (April 2021), and they could be found in the Croatian market in different market channels. The colour of Black Slavonian pig’s fresh meat, which can range from dark to light red, is an important intrinsic quality characteristic, since this breed is known for its distinctive red meat colour. In order to illustrate visual product appearance in the survey, pictures were used. The geographical information attribute includes three levels: continental Croatia, continental Croatia + PDO, and other regions. Continental Croatia + PDO represents the traditional breeding region of Black Slavonian pigs. The rearing area is defined by the PDO product specification, and only meat from pigs reared in that part of Croatia in a specific production system can obtain a PDO label.

In this research, participants were exposed to one of two treatment options in an online survey designed to examine preferences and WTP for fresh meat of the Black Slavonian pig. Prior to the online choice experiment, participants were randomly assigned to either control or information treatment. The control treatment did not include any additional text, only choice experiment instructions. Information treatment was used to inform participants about the rearing systems of Black Slavonian pigs and the impact of the traditional system (outdoor and semi-indoor) on animal welfare and the environmental effect regarding the conventional system (intensive production system—indoor). Using information treatment allows us to evaluate whether a good explanation of the production system and the main characteristics of the Black Slavonian pig could positively affect consumers’ choices. Additionally, the information treatment gave information to the participants regarding each attribute and attribute level shown in Table 1. Specifically, the information treatment was framed as shown in the following subsections.

#### 2.1.1. Production System

Outdoor environments comply with animal welfare criteria and environmental protection. This system of rearing allows pigs to express natural behaviour. Animals can move freely and dig the ground, have access to fresh air and natural light and enough space to explore the environment, and to enjoy free movement and social contact, thus reducing aggression and anxiety.

The semi-indoor production system is also considered a system that respects animal welfare, and it is in line with environmental protection, similar to the outdoor environment but in this system animals are indoors, usually on litter, with outlets where they can go out.

#### 2.1.2. Meat Colour

The meat of the Black Slavonian pig is darker in colour due to higher number of oxidative types of muscles than in modern breeds.

#### 2.1.3. Geographical Indication

Continental Croatia is a traditional breeding region of the Black Slavonian pig. Black Slavonian pig meat is produced exclusively within the administrative borders of Pannonian and northern Croatia.

Continental Croatia + PDO marks the Black Slavonian pig’s traditional meat production area and the protected designation of origin of Black Slavonian pig meat, which guarantees that it is a high-quality authentic product, manufactured in traditional regions and controlled conditions.

Other regions represent those other regions of the Republic of Croatia in which the Black Slavonian pig breed is not traditionally bred.

Price of the meat: variable from HRK 70.00 (€9.24) to HRK 170.00 (€22.45).

### 2.2. Choice Experiment Design

The final experimental design was structured using the software package Ngene version 1.2.1 [41] to design an efficient or D-optimal design, which minimises the element of the asymptotic variance–covariance matrix [42]. Panel mixed logit model with D-error measure was applied, and effect coding was used in the design and model. Effect coding implies that the effect of attribute levels is uncorrelated with a constant term for the status quo alternative [43]. In effect, when coding L, −1 variables are created for one qualitative level, the effect coded variable is set to 1 when the qualitative level is present, equal to −1 when the arbitrary reference level is present and equal to 0 otherwise [43,44]. The following utility function was used in the design:

U = f {Price, Colour, Geographical information, ε},(1)

where attribute Price is the price for one kilogram of fresh pork ham without bones introduced as a metric variable, and Colour and Geographical information are effect-coded attributes. Taken the value +1 if the product is darker red colour and produced in continental Croatia with PDO label, −1 if the product is lighter red colour and produced in other regions of Croatia, and 0 otherwise.

**Figure 1:**
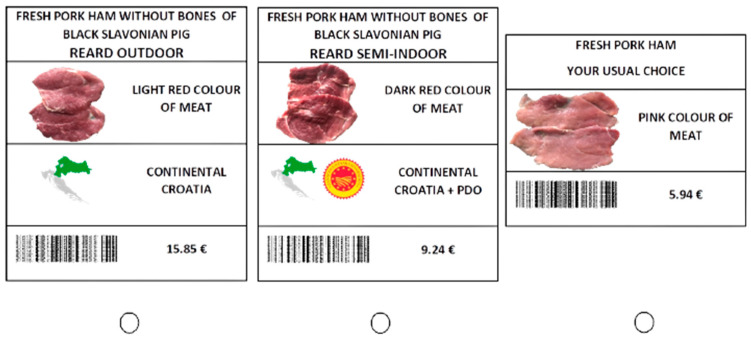
Example of a choice task.

Efficient designs were constructed in the Ngene software by defining a panel mixed multinomial logit model, including alternative specific constants (ASCs), priors (expected values of the βs) derived from pilot studies, and simulation based on the modified Federov algorithm with 500 Halton draws. In order to create a D-efficient design, it was necessary to obtain priors for each attribute and to do so, two pilot studies (*n* = 69 and *n* = 100) were conducted. The design approach presented in Zwerina et al. [45], Bliemer et al. [42], Scarpa et al. [46], Jaeger and Rose [47] was used in the first phase. The pilot study was based on a more basic experimental design, where all parameter priors were assumed to be zero since no prior information existed. Each participant in the pilot study responded to twelve choice situations, and the β coefficients resulting from the multinomial logit model (MNL) estimation of the first pilot data provided priors for the second pilot study. The data from the pilot studies were used to improve the questionnaire’s clarity and test the model specifications. According to Johnson et al. [48], model identification is an important step in constructing a choice experiment because it gives the researcher the ability to obtain unbiased parameter estimates from data for every parameter in the model and ensures the sufficient degree of freedom available for estimation of a choice model. Data from the first pilot study gave information on the parameter distribution used to generate a second efficient design (random parameter). This means that, during the actual choice experiment, the pilot participants are used to estimate the final model and create a new design depending on the new parameter estimates [45]. Data from the second pilot study were used to estimate an error component random parameter logit model (RPL-EC) the coefficient estimates of which were used as random priors to generate the final random parameter design. The final design had a D-error of 0.04636 and included 24 choice tasks in total. However, in order to reduce participants’ fatigue [49], the design was split into two blocks of 12 choices. Participants were randomly assigned to one of the blocks. Each choice task was composed of two labelled product alternatives (Black Slavonian pig reared outdoors and Black Slavonian pig reared semi-indoors) and status quo (constant) alternative (fresh pork ham pink colour and with price €5.97). Labelled choice experiments (alternative specific) were designed to explore different configurations of two or more named alternatives in which the name given to each alternative carried meaningful information for decision-makers [47]. A useful feature of alternative specific designs with labelled alternatives is that they simultaneously create both alternative and choice questions [48]. Figure 1 shows an example of a choice task included in the choice experiment.

### 2.3. Survey Procedure

The survey was conducted using online software as this has advantages over face-to-face, telephone or postal data collection, such as covering a wider geographical area, quick response time, lower cost and fewer errors [34,44,50]. However, there are also some disadvantages to using online surveys, including the availability of computers and internet access, problems with sample collection and sample representativeness, non-response bias, and the fact that online participants tend to read questions more quickly and are more likely to be inpatient [44,51]. To rule out possible sample bias in this study, a post hoc analysis of response time or page timing was conducted. Following Vecchio et al. [52], a short completion and response time suggests that respondents were likely inattentive during the online survey, meaning that respondents probably answered the questions randomly. Therefore, respondents who completed the survey in less than five minutes were removed from the sample.

Participants were recruited from the Qualtrics representative access panel in June 2021, and the survey was distributed by online survey company Qualtrics Inc. Orme [53] recommends a minimum sample size of 200 respondents for studies involving an analysis of differences between sample segments, or 300 respondents if no such analysis is conducted, while sample sizes of 300–500 respondents may be sufficient to obtain valid estimates of stated preferences, depending on the complexity of the experimental design and the type of model used [44,54]. In terms of descriptive statistics, we calculated a required sample size of 384, needed for a 5% confidence interval to estimate a dichotomous variable with a 50% proportion in the population, with a confidence level of 95.0%. In total, 410 Croatian consumers correctly completed the survey, which allows a 4.84% confidence interval. The following criteria were used to select consumers: gender, age and geographical region of the Croatian population. Consumers were randomly invited to participate in a closed panel until the quotas for gender, age and geographical regions were reached. Prior to participation, participants were asked three screening questions about age (at least 18 years old), pork consumption and whether they were responsible (at least in part) for food purchases in their household. After participants passed the screening questions, the choice questions were completed. Participants were asked to select the preferred option among three different fresh pork ham products from Black Slavonian pig and hybrid pig breeds representing the status quo option and the production system treated as an alternative specific attribute. Each respondent scored 12 choice tasks in two treatments, and the Qualtrics software ensured that two blocks of 12 choice questions were presented to the same number of respondents (*n* = 205 per block). The final survey took 15 min to complete.

### 2.4. Estimation Procedure

Descriptive statistics analysis was used to describe the sociodemographic characteristics of the sample, and K-means cluster analysis was used to cluster sociodemographic characteristics of Croatian consumers into three different clusters. The data were analysed using the IBM SPSS Statistics V26 statistical software package. The choice experiment data allowed the estimation of a multinomial logit model (MNL), a random parameter logit model (RPL), and an error component random parameter logit model (RPL-EC) using the following R (version 4.0.2) packages: mlogit [55], gmnl [56], and the lmtest [57].

All models were estimated from 4920 choices made by 410 respondents who have individually performed 12 choice tasks. Exploratory variables included in the model were divided into (1) main effect variables, (2) information treatment variables and (3) clustered sociodemographic variables. The main effect variable includes the price and the alternative specific attributes (colour and geographical indicator), and all variables except the price were effects-coded. Concerning the selected attributes and levels for the RPL-EC model, the utility function for the main effect variable is specified as follows:

(2) $$U_{\mathrm{ijs}}=\mathrm{ASC}+\beta_{1}{\mathrm{PRICE}}_{\mathrm{ijs}}+\beta_{2}{\mathrm{COL}}_{\mathrm{ijs}}+\beta_{3}{\mathrm{GEO}}_{\mathrm{ijs}}+\mu_{i}Z_{\mathrm{ijs}}+\varepsilon_{\mathrm{ijs}}.$$

Model specification for main effects, interaction term information treatment and clustered sociodemographic variable is specified as follows:

(3) $$\begin{array}{l} U_{\mathrm{ijs}}=\mathrm{ASC}+\beta_{1}{\mathrm{PRICE}}_{\mathrm{ijs}}+\beta_{2}{\mathrm{COL}}_{\mathrm{ijs}}+\beta_{3}{\mathrm{GEO}}_{\mathrm{ijs}}+\beta_{4}{\mathrm{COL}}_{\mathrm{ijs}}\times {\mathrm{TreatmentINFO}}_{i}\\ \;\;\;\;\;\;\;+{\;\beta }_{5}{\mathrm{GEO}}_{\mathrm{ijs}}\times {\mathrm{TreatmentINFO}}_{i}+\beta_{6}{\mathrm{COL}}_{\mathrm{ijs}}\times {\mathrm{CluterSD}}_{i}\\ \;\;\;\;\;\;\;+{\;\beta }_{7}{\mathrm{GEO}}_{\mathrm{ijs}}\times {\mathrm{CluterSD}}_{i}+{\;\varepsilon }_{\mathrm{ijs}}. \end{array}$$

By this means, the ASC is an alternative-specific constant identifying the type of meat that is, (a) fresh boneless ham from BSP reared outdoor, (b) fresh boneless ham from BSP reared semi-indoor, versus fresh boneless ham from regular pig produced in a conventional production system (intensive—indoor), the latter being the status quo alternative. PRICE_ijs_ represents the price of 1 kg of fresh boneless pork ham (gluteus medius) of the alternative j, COL_ijs_ represents the fresh meat colour (dark, light) of the alternative j, GEO_ijs_ represents a geographical information (continental Croatia, continental Croatia + PDO, and other regions) of the alternative j, COL_ijs_ and GEO_ijs_ × TreatmentINFO_i_, COL_ijs_ and GEO_ijs_ × ClutserSD_i_ (interaction terms) are colour and geographical information variables interacted with information treatment and sociodemographic clusters, Z_ijs_ is a normally distributed zero mean error component, set to 0 in the utility of the status quo alternative [58], and the ε_ijs_ is the error term. In the random parameter logit (RPL) model and in the error component random parameter logit model (RPL-EC) model, all of the main effect variables except price were modelled as random parameters and assumed to be distributed normally. The RPL and RPL-EC models were estimated considering a panel data structure and using 1000 and 100 Halton draws for simulation. The data were primarily analysed using the baseline multinomial logit model (MNL). In summary, MNL, RPL and RPL-EC models with interaction terms (information treatment and sociodemographic cluster) were estimated.

Willingness to pay is calculated as the difference in Euros (€) between what the consumers are willing to pay for a particular attribute level in comparison with the baseline reference level. Average willingness to pay (WTP) for alternative specific constant and each attribute level of geographical information was calculated as follows:

(4) $${\mathrm{WTP}}_{i|\mathrm{ref}}=-\left(\beta_{i}-\beta_{\mathrm{ref}}\right)/{\;\beta }_{\mathrm{price}}$$

where β_i_ represents the main effect parameter of the attribute i, β_ref_ represents the main effect parameter of the reference level of the attribute, and β_price_ is the coefficient for Price.

## 3. Results

### 3.1. Characteristics of the Sample

A total of 410 respondents completed the survey and the selected sociodemographic variables are presented in Table 2.

The sample consisted of 51.0% female and 49.0% male respondents. Overall, 41% of the respondents were between 25 and 44 years old, and 26.8% of the respondents were 55 or older. Respondents were predominantly from urban areas (78.5%) and not associated with agriculture (55.6%). Respondents were evenly distributed by geographic region. At the time of the study, the majority of respondents were employed (in the private, public or civil sector) (62.0%), and 75.4% of them held a graduate university degree (university or professional studies) or a post-graduate university degree (Master’s or PhD), and 24.6% of them had a secondary school degree or lower (primary or high school diploma). The higher proportion of respondents with higher levels of education in the sample could be due to the fact that the responses were collected online and the respondents were mostly from urban areas, which is common for cities. The results obtained are in line with the Croatian census [59], according to which 22.9% of the Croatian population in urban areas have a high level of education, while in rural areas only 8.5% of the population has a high level of education. Respondents mostly live in families with 2–4 household members (82.7%), with no children under 15 years of age (68.8%) and with no elderly person over 60 years of age (63.4%) in the household. Respondents reported that their monthly household income is average (36.6%), which is consistent with data from the Croatian Statistical Office [60].

### 3.2. Models Estimation and Interaction of Information Treatment with Main Effect Variables

The parameter estimates for the main effect variables and the interaction terms between the main effect variables and the information treatment are presented in Table 3. The first column illustrates the results for the baseline MNL model, and the second and third columns represent the RPL and RPL-EC models for analysing the preference heterogeneity of Croatian consumers. The log-likelihood ratio test (LR test), the Akaike information criterion (AIC) and the Bayesian information criterion (BIC) were calculated to compare the selected models based on their statistical fit. These measures can be used to describe the relative adequacy of the three models presented. The higher the value of the log-likelihood criterion and the lower the information of the AIC and BIC criteria, the better a model fits the data. An LR test rejects the null hypothesis that all coefficients are equal to zero (*p* < 0.01), and all coefficients of the main effect variables except colour and geographical information (continental Croatia) are significantly different from zero. In all three models, the estimated parameters have the same sign and are similar. Based on the data presented in Table 3, the main observation is that the RPL model is an improvement over the baseline MNL model for the information criteria (AIC and BIC) (9865.369 and 9975.887 in the RPL model compared with a respective 10,214.84 and 10,286.35 in the MNL model). The RPL and RPL-EC models account for preference heterogeneity in consumer preferences and fit the data better than the MNL baseline model. However, all criteria indicate that the RPL-EC model (6588.258; 6724.78) fits the data better than the MNL and RPL models, and the value of the RPL-EC model is closer to 0. Moreover, the standard deviations reflecting the variation between respondents of the RPL-EC model are statistically significant, suggesting that consumer preferences for colour and geographical indicators (continental Croatia and continental Croatia + PDO) are heterogeneous and that the RPL-EC model is appropriate. Continental Croatia + PDO has the highest standard deviations among the proposed attributes. This means that there is heterogeneity among the surveyed consumers with regard to this attribute of fresh meat from the Black Slavonian pig. Non-status quo is an additional error component that captures the correlation between the non-status quo alternatives assuming a normal distribution. Intuitively, researchers might think that hybrid meat from conventionally reared pigs, which is well known to respondents in this study, has lower variance than the other two options, which are less familiar to respondents. For this reason, an error component is added to the function in the model RPL-EC so that the status quo alternative has a separate error term. The standard deviation of the non-status quo alternative is positive and significant, which means that the unobserved heterogeneity associated with the designed alternatives confirms the assumption of a non-constant status quo effect across respondents.

Table 3 shows that fresh boneless ham produced from the meat of the Black Slavonian pig reared outdoors and semi-indoors is preferred over hybrid pig meat obtained from a conventional production system, which represents the status quo option. Consumers have generally obtained a lower utility from the status quo option than from the designed alternatives. The positive and significant alternative specific coefficients for fresh boneless ham produced from the meat of the Black Slavonian pig reared outdoors, and semi-indoors indicate that the unobserved utility associated with the production system utility function is greater than utility received from fresh boneless ham produced in a conventional production system (intensive—indoor). The highest utility increment occurs due to the fresh boneless ham produced from the meat of the Black Slavonian pigs reared outdoors.

The price attribute is negative and highly significant, showing that, in accordance with economic theory, a higher price reduces consumer utility. 

According to the results presented in Table 3, the negative sign of the dark red meat colour is barely significant (*p* < 0.1) and decreases consumer utility. In contrast, the light red meat colour increased consumer utility.

Regarding a geographical indicator, the presence of a label indicating the pigs were reared in continental Croatia + PDO improved the selection probability when compared with the fresh ham of the pigs reared in other regions of Croatia and the fresh ham of the pigs bearing no such label (a status quo option). In general, the participants obtained the greatest utility from a label indicating that the animals were reared in continental Croatia + PDO, while the label indicating they were reared in continental Croatia is not preferred by participants. This could be because Croatian consumers are not yet familiar with the mentioned label continental Croatia but are familiar with the PDO sign which adds extra utility to Croatia’s continental provinces. Still, from the results in Table 3, it can be observed that the continental Croatia label has a positive information treatment sign, which means that this attribute adds extra utility for consumers after they are given information about the meaning of the geographical labels.

The results of the RPL-EC model, presented in Table 3, confirm that providing information about production systems and the production of Black Slavonian pig meat has a positive effect on participants’ perceived utility. This means that, in the information treatment, consumers value Black Slavonian pig meat reared outdoors and semi-indoors more than they do hybrid meat obtained from a pig reared conventionally. Additionally, consumers slightly prefer fresh meat obtained from Black Slavonian pigs reared outdoors than semi-indoors. After being given information, they preferred darker over the light colour of the meat. 

### 3.3. Models Estimation and Interaction of Sociodemographic Clusters with Main Effect Variables

K-mean cluster analysis was used to cluster Croatian consumers into three clusters according to their sociodemographic characteristics. Cluster one (*n* = 159) represents young urban families (YUF) with children, as respondents in this group are 35 to 44 years of age with two to four family members in the household and at least one child younger than 15 years. This group does not have any connection with agriculture, and its monthly household income is between €1518.89 and €2179.08. The second cluster (*n* = 146) consists of older urban families (OUF) (55 and more years), with two to four family members, without younger children, with no connection to agriculture, and a monthly household income between €990.62 and €1254.62. The third group (*n* = 105) represents young rural families (YRF). This group consists of respondents between 25 and 35 years with two to four family members, without children younger than 15 years of age, a connection with agriculture, and a monthly household income of between €726.49 and €990.49.

Table 4 shows interaction terms (sociodemographic clusters) with main effect variables. The intercept values for Black Slavonian pig meat produced outdoors and semi-indoors are all significant but negative, indicating that clusters 2 (OUF—older urban families) and 3 (YRF—young rural families) perceive a lower added value for this product than cluster 1 (YUF—young urban families). Thus, Fresh Black Slavonian pig meat produced both outdoors and semi-indoors receives a more favourable valuation from young urban families. No significant difference between clusters regarding the darker colour of the Black Slavonian Pig meat was observed.

### 3.4. Willingness to Pay Estimation

The average WTP estimations using MNL, RPL and RPL-EC models are presented in Table 5. WTP presented in Table 5 are the maximum premium prices for the attributes compared with the reference product (meat obtained from hybrid pigs reared in conventional production system). Consumers are willing to pay a premium for fresh ham of the pigs reared outdoors and semi-indoors as well as for geographical information labels. Surveyed consumers are willing to pay €6.67/kg more for fresh boneless ham produced from the meat of the Black Slavonian pig reared outdoors and €4.27/kg for fresh boneless ham produced from the same breed reared semi-indoors over the price for regular pork. Consumers are willing to pay €4.23/kg more for fresh meat from the Black Slavonian pig with the continental Croatia + PDO label compared with other regions (regions of the Republic of Croatia in which Black Slavonian pigs are not traditionally reared). Consumers are willing to pay a relatively high premium for fresh ham from Black Slavonian pigs raised outdoors and labeled with a PDO indicating their origin in continental Croatia. Producers can obtain a higher price for this product compared to fresh meat from Black Slavonian pigs raised semi-indoors and labeled only as reared in continental Croatia, which also commands a premium price but to a lesser extent. Thus, the PDO label seems to increase the perceived value of products from Croatia’s continental provinces.

**Figure 2:**
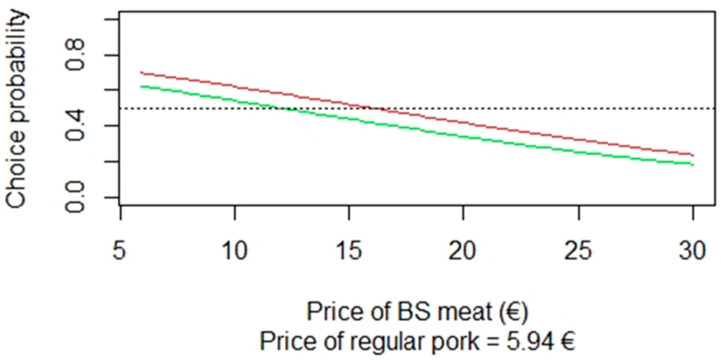
Probability of choosing an option among two alternatives.

The probability that surveyed consumers will buy fresh ham meat obtained from Black Slavonian pigs over regular pork priced €5.94/kg was calculated from the MNL model using the estimated parameters. The probability that consumers will purchase fresh ham with different production methods and labels is presented in Figure 2. The red line in Figure 2 represents a darker red fresh ham meat obtained from the Black Slavonian pig reared outdoors and is labelled as continental Croatia + PDO. The results show that Croatian consumers would select a darker meat obtained from the Black Slavonian pigs reared outdoors and labelled with a PDO sign until its price reached €18.00/kg (Figure 2). At this price level, the probability of choosing the fresh Black Slavonian pig meat over regular pork (i.e., meat from hybrid pigs from conventional production system) is the same. The green line (Figure 2) represents the light red fresh ham meat obtained from the Black Slavonian pigs reared semi-indoor, labelled as continental Croatia. Compared with regular pork for €5.94/kg, consumers have a higher probability of preferring lighter red, fresh ham meat obtained from the Black Slavonian pigs that are reared semi-indoors up to the price of €13.00/kg and that, beyond this price, the purchase of regular pork for €5.94/kg becomes more likely.

## 4. Discussion

Using a discrete choice experiment, Croatian consumers indicated their preferences and WTP for boneless fresh ham from the Black Slavonian pig. According to the results of the choice experiment, Croatian consumers prefer meat from Black Slavonian pig reared outdoors and semi-indoors over meat from hybrid pigs reared conventionally (indoor). These findings confirm previous research. In a pilot study conducted with a smaller sample (100 Croatian consumers), free-range and semi-indoor production systems were rated positively compared with a conventional pig farming system [61]. Consumers also preferred a darker red colour of the fresh meat of the Black Slavonian pig labelled with geographical information, such as continental Croatia or continental Croatia + PDO [61]. Krystallis et al. [62] conducted a conjoint analysis to determine the attitudes of European citizens (Belgium, Denmark, Germany and Poland) towards the pig production system. They concluded that factors such as housing type and flooring, as well as efforts to protect soil, air and water, strongly influenced the evaluation of the pig production system, while farm size, fat and quality had a negative benefit for the respondents. Pugliese and Sirtori [63] studied indigenous pig breeds from southern Europe, which are in high demand due to their better meat quality, and concluded that a free-range system increases the value of animal products. The geographical indicators (continental Croatia and continental Croatia + PDO) had a higher value for consumers than fresh ham from pigs reared in other regions of Croatia and fresh ham from pigs that did not carry such a label (a status quo option). Expected quality can result from a perceived intrinsic perspective based on the evaluation of taste, texture and smell, as well as an extrinsic perspective based on the characteristics of the product such as packaging and price [64]. In the choice experiment study, conducted to investigate Spanish consumers’ preferences for the Iberian cured ham production system, the region where the ham is produced was ranked as the most important, with consumers preferring hams from areas where Iberian cured ham is traditionally produced over other regions of Spain. They also preferred hams from animals reared in the traditional *montanera* production system [64].

In terms of information treatment, consumers clearly preferred the options for fresh Black Slavonian pigs reared outdoors and semi-indoors over meat from hybrid pigs. In addition, the option Black Slavonian pig reared outdoors gave them more value than Black Slavonian pigs reared semi-indoors. Consumers who had more information about the production system preferred the dark red colour of the meat to consumers who did not have this additional information, and, though the observed difference was not significant in the RPL-EC model, it was significant in the MNL model (*p* < 0.01) and in RPL (*p* < 0.1). From the results of this study, it can be concluded that the information treatment improves the validity of the choice experiment. This is consistent with the findings of Khachatryan et al. [31], who used different consequence treatments and a control treatment to investigate the role of trust and consequence perception on consumers’ preferences for products with eco-labels. The authors concluded that consequence treatments serve as a means to improve internal validity in the choice experiment and that the presence of consequence treatments increased WTP compared with the control group. In general, consumers do not know much about the production system of indigenous pig breeds, animal welfare and environmental impacts of production [40]. In the present study, information about the rearing system, meat colour and geographical information enabled consumers to make more informed choices. This is because almost half of the respondents had never tasted Black Slavonian pig meat but nonetheless chose the darker colour of the meat according to the information given. Vitale et al. [65] and Cerjak et al. [66] found that sensory characteristics and information about indigenous pig breeds influenced consumers’ preferences and shifted their expectations towards the informed test. Dransfield et al. [67] studied consumers’ reactions to the appearance and taste of pork with and without information about free-range pigs in France, Denmark, Sweden and the UK. In all four of the studied countries, consumers were mainly oriented towards the colour and fattiness of the meat, and then towards the marbling and the drip. The colour differed significantly between the countries studied. French consumers preferred a darker colour, while British and Danish consumers preferred a lighter colour of meat; all consumers preferred lean meat to fatty meat. García-Gudiño et al. [40] investigated Spanish consumers’ perceptions of different aspects of pig production and animal welfare using conjoint analysis and segmented consumers according to their knowledge of Iberian pig production. The authors concluded that consumers know little about Iberian pig production, but that they prefer Iberian products, especially when the animals are reared freely and under natural conditions and they attach great importance to animal welfare. Consumers considered Iberian products to be of higher quality, tastier, healthier and produced with higher animal welfare standards than pork from white cosmopolitan pig breeds [40]. On the other hand, they believed that Iberian products were more expensive, although this depended on their level of knowledge about the Iberian production system and the products of this breed. The results of this study indicate that consumers’ knowledge about the meat of indigenous pig breeds (Black Slavonian pig) needs to be improved. This is in line with the results of the previously mentioned study [40], in which authors found that consumers who know about Iberian pork products and their premium price place a higher value on them; however, knowledge about the breed and the labelling of the products is essential. The authors stressed the need to clearly indicate information about the breed and rearing conditions on the label, which was very important for the consumers studied in both studies. In terms of sociodemographic clusters, younger urban families give a more positive evaluation of fresh Black Slavonian pig meat and appreciate more than consumers in other clusters the darker red colour of fresh Black Slavonian Pig meat labelled continental Croatia + PDO. Previous studies have investigated the role of drip, colour, marbling and fat cover as intrinsic cues that determine consumers’ purchasing decisions [68]. The results of the study show that consumers preferred pork without fat cover. Preference for colour was the same for all respondents, but consumers over 35 preferred darker meat, while respondents’ sociodemographic characteristics did not affect the preference for marbling and dripping. In addition, the authors concluded that taste was the most important sought-after feature when buying pork, while convenience was second, and price, availability and nutritional values were of lesser importance [68].

The study results indicate that the consumers surveyed are willing to pay a premium for the fresh meat of Black Slavonian pigs reared outdoors and semi-indoors and with geographical labelling compared with the meat of hybrid pigs from conventional rearing. When buying pork, consumers are confronted with different alternatives, and their choice will be shaped by the information available to them and the alternatives available. Consumers are willing to pay the large premium for fresh ham from Black Slavonian pigs reared outdoors and labelled “reared in continental Croatia + PDO” compared with fresh meat from semi-indoor reared Black Slavonian pigs labelled “reared in continental Croatia”. The results of the study show that the PDO label brings added value to the continental provinces of Croatia. This is in line with the study by Lusk et al. [69], which found that consumers from the United States are, on average, willing to pay a premium price for pork products that have good public characteristics, such as products with certifications related to the environment, animal welfare and antibiotics. In the study by Sahelices et al. [39], a high percentage of the consumers studied in a choice experiment rated the PDO label positively (93.6%) and were willing to pay a price premium of more than 10.0% for dry-cured Iberian ham. Many European consumers have greater confidence in local food and assume that it is of better quality, but this also depends on the country. The preference for local food is higher in Germany and Norway than in the UK [70]. Dransfield et al. [67] came to the same conclusion when they studied consumers from four European countries (France, Denmark, Sweden and the UK) who preferred pork labelled as locally sourced to imported and that labelled as pork from pigs reared outside as opposed to in-side. Consumers in this study were also willing to pay 5% more for the label “from their own country” and “reared outside” [67]. The study results show that there is label potential in the Croatian market, which is in line with the findings of Marescotti et al. [71]. The authors found a similar situation in Italy, where consumers prefer to choose meat with the protected geographical indication (PGI) label rather than the new label, probably because they are better informed about it, but their preferences for the new label were heterogeneous, as this study also shows. Therefore, it is important to inform Croatian consumers about the meaning of the continental Croatia and continental Croatia + PDO labels and why fresh meat with these labels has a higher price than normal fresh pork or meat from Black Slavonian pigs from other regions. The aforementioned quality labels add value to the product as they identify fresh meat from Black Slavonian pigs from traditional rearing regions and under controlled conditions and guarantee consumers that it is a high-quality authentic product.

Before drawing conclusions, we should mention some limitations of the study. Future research should focus on a larger sample of respondents and use other survey methods and modelling approaches. For instance, non-hypothetical choice experiments with actual purchases can be used to confirm and improve our results.

## 5. Conclusions

When assessing the preferences of Croatian consumers in relation to the rearing system, meat colour and geographical information through a choice experiment, it can be seen that consumer preferences are heterogeneous. In Croatia, the certification of fresh meat from indigenous pigs is not regulated and consumers obtained higher level of utility for a PDO-labelled product compared with a product labelled as continental Croatia only, probably because consumers know more about the PDO label. It is important to create policies and tools for improving meat production standards and sharing information through labels and declarations. Therefore, producers, retailers and policy makers need to work together and play an important role in providing consumers with the right information about the production system, labelling and meat characteristics from indigenous pig breeds. The Croatian market should introduce labels indicating that the animals were reared in continental Croatia or reared in continental Croatia + PDO. These labels should only be able to be acquired by producers who produce the meat of the Black Slavonian pig according to the specification. The creation of marketing opportunities in rural areas through the development of a brand and a label that conveys biodiversity, sustainability and ecological values is also important for producers, as they can obtain a premium price and additional profit.

## Figures and Tables

**Table 1:** Attributes and levels used in the choice experiment on fresh pork ham without bones.

Product Attribute	Product Level	
Price	HRK 70.00 (€9.24)	
	HRK 120.00 (€15.85)	
	HRK 170.00 (€22.45)	
Colour	dark red	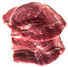
	light red	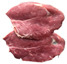
Geographical information	Continental Croatia	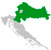
	Continental Croatia + PDO	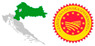
	Other regions	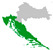

**Table 2:** Main descriptive statistics of the respondents’ sociodemographic characteristics.

Characteristics		Percent of Total (%)		
		Total Sample (410)	Control Group (204)	Information Group (206)
Gender	Male	49.0	50.5	47.6
	Female	51.0	49.5	52.4
Age	18–24	13.4	14.2	12.6
	25–34	20.5	20.1	20.9
	35–44	20.5	20.1	20.9
	45–54	18.8	20.6	17.0
	>55	26.8	25.0	28.6
Place of residence	Urban	78.5	80.4	76.7
	Rural	21.5	19.6	23.3
Connection with agriculture	No	55.6	56.4	54.9
	Yes	44.4	43.6	45.1
Region	Central Croatia	24.9	26.5	23.3
	North-western Croatia	24.6	24.5	24.8
	Eastern Croatia	21.5	19.6	23.3
	Northern Adriatic and Lika	15.9	17.2	14.6
	Middle and South Adriatic	13.2	12.3	14.1
Labour situation	Student	11.0	12.3	9.7
	Unemployed	8.0	8.8	7.3
	Employed part-time	4.1	5.4	2.9
	Employed	62.0	58.3	65.5
	Retired	14.9	15.2	14.6
Education	High school and lower education	24.6	24.5	24.8
	Higher education (university or professional studies, e.g., bachelor’s)	50.5	52.9	48.1
	University degree (master’s and/or PhD)	24.9	22.5	27.2
Number of household members	1	6.3	3.4	9.2
	2–4	82.7	85.8	79.6
	5–8	11.0	10.8	11.2
Number of children below 15 years in the household	0	68.8	65.7	71.8
	1	17.1	17.2	17.0
	≥2	14.1	17.2	11.2
Household monthly income (€)	0.00–462.23	4.6	3.4	5.8
	462.36–726.36	6.6	6.4	6.8
	726.49–990.49	18.3	24.0	12.6
	990.62–1254.62	16.8	13.2	20.4
	1254.75–1.518.89	14.4	12.7	16.0
	1518.89–2179.08	22.2	21.6	22.8
	2179.21–2707.34	12.4	14.7	10.2
	>2707.34	4.6	3.9	5.3

**Table 3:** Estimated parameters of the MNL, RPL, and RPL-EC models and interaction of information treatment with main effect variables.

Mean Estimates	Coefficients		
	MNL	RPL	RPL-EC
BS reared outdoors	0.4561 ***	0.4003 ***	1.0255 **
BS reared semi-indoors	0.3709 ***	0.3195 ***	0.6570 *
Price	−0.0109 ***	−0.0130 ***	−0.0203 ***
Colour (dark)	−0.0573 .	−0.0877 .	−0.0536
Geographical Information			
Continental Croatia	−0.0121	−0.0183	−0.0346
Continental Croatia + PDO	0.2303 ***	0.2832 ***	0.3427 ***
Information treatment interaction with main effect variables			
BS reared outdoors × Information	0.2924 ***	0.3435 ***	1.2164 *
BS reared semi-indoors × Information	0.2374 ***	0.2573 ***	1.1162 *
Colour (dark) × Information	0.1042 **	0.1282 .	0.0999
Geographical Information			
Continental Croatia × Information	0.0564	0.0783	0.1310
Continental Croatia + PDO × Information	0.0112	0.0305	0.0109
St dev. of mean estimates (std. err.)			
Colour (dark)		0.5615 ***	0.4759 ***
Geographical Information			
Continental Croatia		0.4679 ***	−0.5173 ***
Continental Croatia + PDO		0.4261 ***	0.4866 ***
Non status quo			5.1799 ***
Number of respondents	410	410	410
Number of observations	4920	4920	4920
LR test	−5096.4	−4915.7	−3273.1
AIC	10,214.84	9865.369	6588.258
BIC	10,286.35	9975.887	6724.78

Note: MNL—multinomial logit, RPL—random parameter logit, RPL-EC—error component random parameter logit, LR test—log likelihood ratio test, AIC—Akaike information criteria, BIC—Bayesian information criteria, BS—Black Slavonian pig meat, PDO—protected designation of origin. Significance: . *p* < 0.1, * *p* < 0.05, ** *p* < 0.01, *** *p* < 0.001.

**Table 4:** Estimated parameters of the MNL and RPL models and interaction of sociodemographic clusters with main effect variables.

Mean Estimates	Coefficients	
	MNL	RPL
BS reared outdoors	1.0490 ***	1.0372 ***
BS reared semi-indoors	0.8755 ***	0.8442 ***
Price	−0.0110 ***	−0.0130 ***
Colour (dark)	0.0151	−0.0046
Continental Croatia	0.0147	0.0084
Continental Croatia + PDO	0.2754 ***	0.3318 ***
Sociodemographic cluster interaction with main effect variables		
BS reared outdoors × OUF	−0.7634 ***	−0.7813 ***
BS reared semi-indoors × OUF	−0.7048 ***	−0.7117 ***
BS reared outdoors × YRF	−0.6274 ***	−0.6469 ***
BS reared semi-indoors × YRF	−0.4663 ***	−0.4793 ***
Colour (dark) × OUF	−0.0750	−0.0677
Colour (dark) × YRF	0.0270	0.0245
Continental Croatia × OUF	0.0701	0.0970
Continental Croatia + PDO × OUF	−0.1516 *	−0.1538 .
Continental Croatia × YRF	−0.0740	−0.0553
Continental Croatia + PDO × YRF	0.0377	0.0599
St. dev. of mean estimates (std. err.)		
Colour (dark)		0.5476 ***
Geographical Information		
Continental Croatia		0.4128 **
Continental Croatia + PDO		0.4061 ***
Number of respondents	410	410
Number of observations	4920	4920
LR test	−5043.2	−4868
AIC	10,118.49	9780.051
BIC	10,222.51	9923.074

Note: MNL—multinomial logit, RPL—random parameter logit, LR test—log likelihood ratio test, AIC—Akaike information criteria, BIC—Bayesian information criteria, BS—Black Slavonian pig meat, PDO—protected designation of origin, OUF—older urban families, YRF—young rural families. Significance: . *p* < 0.1, * *p* < 0.05, ** *p* < 0.01, *** *p* < 0.001.

**Table 5:** Willingness to pay (€/kg) for alternative specific constant and geographical information attribute compared with reference point.

Attributes	WTPMNL	WTPRPL	WTPRPL-EC
BS reared outdoors cf. regular pork	7.29	4.11	6.67
BS reared semi-indoors cf. regular pork	5.91	3.31	4.27
Continental Croatia cf. other regions	3.27	4.81	1.78
Continental Croatia + PDO cf. other regions	5.92	6.31	4.23

Note: WTP—Willingness to pay, MNL—multinomial logit, RPL—random parameter logit, RPL-EC—error component random parameter logit, BS—Black Slavonian pig meat, PDO—protected designation of origin, cf.—the comparison is made with regular pork (meat obtained from hybrid pigs reared conventionally) and other regions (regions of the Republic of Croatia in which Black Slavonian pigs are not traditionally bred).

## Data Availability

The data are available from the corresponding author.
